# Erdheim-Chester病的突变谱系及治疗选择

**DOI:** 10.3760/cma.j.issn.0253-2727.2023.10.019

**Published:** 2023-10

**Authors:** 敏 郎, 道斌 周, 欣欣 曹

**Affiliations:** 中国医学科学院、北京协和医学院北京协和医院血液内科，北京 100730 Department of Hematology, Peking Union Medical College Hospital, Chinese Academy of Medical Sciences & Peking Union Medical College, Beijing 100730, China

Erdheim-Chester病（Erdheim-Chester disease, ECD）是最常见的非朗格汉斯细胞组织细胞增生症（LCH），1930年由Jakob Erdheim和William Chester首次报道，目前全球有约1 500例已知病例。ECD高发年龄为50～70岁，男性略多，主要累及骨骼、肺、腹膜后、心血管、中枢神经系统、垂体、眶后组织及皮肤等。骨骼受累者主要表现为双侧长骨骨干和干骺端对称性骨皮质硬化，仅50％的患者存在骨痛。胸膜或肺实质受累者可表现为呼吸困难、咳嗽等，肺功能可显示限制性通气障碍和弥散功能减低，胸部CT可表现为胸膜增厚、胸腔积液、小叶中央结节影、磨玻璃影或肺囊肿等。腹膜后纤维化患者可因肾周脂肪和肾周筋膜对称浸润出现“毛肾”现象，病变可包绕输尿管造成输尿管狭窄、肾积水、慢性肾功能不全。大血管受累患者病变可累及主动脉干分支，可出现腹痛、胸痛、背痛。ECD可累及心脏，右心房或房室沟占位最常见。中枢神经系统受累是不良预后因素，临床表现受病灶部位影响，不同部位病变有不同的临床表现。垂体受累导致的尿崩症是ECD患者最常见的内分泌表现。部分患者会出现眶后组织增生或眼睑黄色瘤。ECD的诊断需要依靠特征性影像学及组织病理学表现。若患者有不明原因骨痛（特别是四肢远端骨痛），伴心血管、神经系统或皮肤表现，同时骨显像提示长骨骨干和干骺端对称性骨皮质硬化，应怀疑ECD。ECD的病理表现为受累脏器中有泡沫样组织细胞，伴炎性细胞和多核巨细胞（Touton细胞）浸润，纤维组织混合其中或包绕在外，免疫组化：CD68（+）、XⅢa（+）、CD163（+）、Fascin（+）、CD1a（−）、langerin（−）、S100（+/−）。2012年在ECD病灶组织中发现了BRAF^V600E^突变，从而确定ECD为一种克隆性肿瘤；RNA测序发现ECD具有与髓系祖细胞或巨噬细胞类似的转录谱，且有研究在BRAF^V600E^突变阳性ECD患者的CD34^+^造血干/祖细胞、单核细胞和髓系树突状细胞中也发现了BRAF^V600E^突变，提示ECD是一种髓系来源的肿瘤；还有研究发现，ECD患者IL-6、INF-α等细胞因子和趋化因子水平升高，这些因子可能发挥了组织细胞的局部激活和募集作用，进而导致组织损伤和器官功能障碍，因此目前认为ECD是一种炎性髓系肿瘤。ECD的一线治疗包括干扰素-α（IFN-α），BRAF抑制剂、MEK抑制剂等。本文回顾了ECD相关文献，对ECD的突变谱系及治疗选择进行综述。

一、突变谱系

自2012年首次在ECD患者病变组织中发现BRAF^V600E^突变以来，研究发现多数ECD患者携带基因突变和（或）基因融合，主要涉及MAPK（RAS-RAF-MEK-ERK）通路和PI3K-AKT-mTOR通路，BRAF^V600E^突变导致MAPK通路过度激活，是ECD发病的驱动突变，证明ECD是克隆性肿瘤性疾病，同时为ECD的分子治疗提供了靶点。

1. MAPK通路：

（1）BRAF突变：BRAF^V600E^是MAPK通路最常见的突变，在黑色素瘤、结直肠癌、甲状腺癌、毛细胞白血病中均有报道。2012年，Blombery等[1]首次在ECD患者病变部位活检组织中发现了BRAF^V600E^突变，为ECD是克隆性肿瘤性疾病提供了证据，也为BRAF抑制剂靶向治疗ECD提供了依据。一项纳入24例ECD患者的研究证明，BRAF^V600E^的突变频率约为54％，该研究还通过免疫组化证实ECD患者的泡沫样组织细胞是存在突变的细胞。其他多项研究报道，BRAF^V600E^的突变频率为50％～69.2％。2016年我中心回顾了16例ECD患者，应用PCR和免疫组化检测的BRAF^V600E^的突变频率分别为68.8％和50％，与国际报道的突变频率相似[2]。2022年我中心在16例ECD患者的循环游离DNA（cfDNA）中测得BRAF^V600E^的突变频率为62.5％，与在组织中的比例高度一致，证明cfDNA可以作为组织测序的可靠补充[3]。研究还发现BRAF^V600E^突变与中枢神经系统、心脏及冠状动脉受累相关，与死亡率高也相关。

在LCH合并ECD患者中也发现有BRAF^V600E^突变，证明LCH和ECD的关联与BRAF^V600E^突变相关。2017年一项研究在3例LCH合并ECD患者中发现2例携带BRAF^V600E^突变。2022年我中心通过二代测序发现5例LCH合并ECD患者全部携带BRAF^V600E^突变[4]。还有病例报道显示，BRAF^V600E^突变阳性的ECD合并皮肤LCH患者同时合并甲状腺乳头状癌。

另外，还有研究在ECD患者中检测到BRAF^L485W^突变[5]、BRAF^R603Q^突变[6]、BRAF^T599_V600delinsRE^突变[7]、RNF11-BRAF融合及CLIP2-BRAF融合[6]。

（2）ARAF突变：2015年一项研究对14例ECD患者的新鲜冷冻标本进行了全外显子测序，首次发现有的ECD患者携带ARAF突变[6]。2017年的一项研究纳入60例ECD患者，发现1例（1.67％）携带ARAF^D228V^突变[8]。同年还报道了1例ECD眼部受累患者ARAF^S214A^突变阳性。2018年Ozkaya等[9]对38例ECD患者进行了基因突变检测，发现1例ARAF^A225V^突变和1例ARAF^S214A^突变。

（3）NRAS、KRAS突变：2013年Diamond等[10]首次报道了1例BRAF野生型ECD患者携带NRAS^Q61R^突变，进一步证明ECD由MAPK通路激活驱动，同时提示MEK抑制剂可用于ECD的治疗。另外两项研究测得的NRAS突变频率分别为3.75％（3/80）[11]和7.14％（1/14）[6]。2017年Lee等[12]发现1例ECD患者同时存在NRAS和BRAF突变。2018年Ozkaya等[9]对38例ECD患者进行了靶向测序，发现1例NRAS^Q61R^突变，同时还发现1例KRAS^G12S^突变，这是文献首次报道ECD患者携带KRAS突变。2019年Janku等[5]在1例ECD患者病变组织中发现NRAS^Q61R^突变，还在另1例ECD患者的cfDNA中发现同时存在BRAF^V600E^和KRAS^G12R^。有个案报道显示NRAS突变阳性的ECD患者合并急性髓系白血病[13]，也有文献报道KRAS突变阳性的ECD患者合并慢性粒-单核细胞白血病（CMML）[14]，提示NRAS、KRAS突变的ECD患者可能更容易合并其他血液系统恶性肿瘤。

（4）MAP2K1突变：MAP2K1是BRAF野生型ECD患者最常见的突变，一般仅在BRAF^V600E^野生型患者中发现。2015年一项研究对14例ECD患者的新鲜冷冻标本进行了全外显子测序，首次发现2例（14％）携带MAP2K1突变[6]。Lee等[12]测得的MAP2K1突变频率为15.4％（2/13），Ozkaya等[9]测得的MAP2K1的突变频率为15.8％（6/38）。我中心对45例ECD患者进行二代靶向基因测序，发现有4例（8.9％）携带MAP2K1突变[4]。

除MAP2K1突变外，还有研究发现有ECD患者携带MAP3K1突变[12]、MAP1K2突变[9]、MAP2K2突变[5]。

2. PI3K-AKT-mTOR通路：PI3K亚型包括PIK3CA和PIK3CD，是PI3K-AKT信号通路的一部分，在细胞增殖和存活等多种细胞功能中发挥作用。2014年一项研究在55例ECD患者中检测到7例（12.7％）携带PIK3CA突变，其中4例同时携带BRAF突变，证实了PI3K-AKT-mTOR途径激活在ECD中的作用[11]。2015年Diamond等[6]对18例BRAF野生型ECD患者的新鲜冷冻标本进行靶向测序，发现PIK3CA的突变频率为17％（3例）。2018年Ozkaya等[9]发现1例ECD患者同时具有MAP2K1突变和PIK3CA突变。2022年我中心在16例ECD患者的组织中发现1例（6.25％）PIK3CD突变阳性，该例患者的cfDNA中也携带PIK3CD突变[3]。

3. 其他突变：2007年发现1例ECD患者携带t（12;15;20）（q11;q24;p13.3）染色体平衡异位，这是文献中首次报道ECD的遗传学改变[15]。2016年报道了1例肝移植术后肝脏、骨骼病理诊断为ECD的患者体细胞存在PDGFRA、PTEN和HNF1A突变[16]。2017年，Lee等[12]在13例ECD患者病变组织中发现3例携带ASXL1突变。同年，一项纳入60例ECD患者的研究发现1例KIF5B-ALK融合[8]。2019年，一项研究对28例ECD患者的病变组织进行二代测序，发现3例ASXL1突变、1例U2AF1突变、1例MITF扩增、1例SOX2扩增、1例LMNA-NTRK1融合。该研究还在1例ECD合并Rosai-Dorfman病（RDD）患者的组织中检测到MIR143HG-NOTCH2融合[5]。2021年，另一项研究在36例ECD患者骨髓中检测到11例TET2、2例ASXL1、2例DNMT3A和22例U2AF1突变[17]。2022年，我中心对45例ECD患者的病变组织进行二代测序，发现5例携带髓系突变，分别为TET2、ASXL1、GATA2、DNMT3A和JAK2[4]。同年还报道了1例ECD患者同时存在BRAF^V600E^和BCOR突变[18]，1例ECD患者携带CSF1R体细胞突变[19]。

研究发现ECD患者可合并其他髓系肿瘤，有研究报道了3例ECD合并CMML患者，发现他们携带KRAS、NRAS、ASXL1和DNMT3A突变[14]。Papageorgiou等[20]报道了1例BRAF^V600E^突变阳性的ECD患者，在ECD诊断15个月后诊断了NPM1^+^/FLT3^+^急性髓系白血病（AML）。

二、治疗

1. IFN-α及聚乙二醇化IFN-α：IFN-α是ECD的一线治疗方案，有效率为50％～80％，应用剂量为300～900万单位，每周3次，起效时间3～6个月，应使用至少2～3年，50％的患者会出现疲劳、抑郁和血细胞减少等不良反应。2011年一项研究纳入了53例ECD患者，其中46例患者接受了IFN-α或聚乙二醇化IFN-α（PEG-IFNα）治疗，其中35％接受常规剂量的IFN-α（<600万单位，每周3次）或PEG-IFNα（<180 µg/周），65％接受高剂量治疗，结果显示，高剂量IFN-α对心脏或中枢神经系统受累的ECD患者效果更好[21]。2019年我中心回顾性分析了接受高剂量IFN-α治疗的32例ECD患者，总体临床有效率为80.0％[22]。推荐中枢神经系统和心血管系统受累的患者应用高剂量IFN-α治疗。与IFN-α相比，患者对PEG-IFNα的耐受性更好，标准剂量为135 µg/周，若有中枢神经系统或心脏受累，可增加剂量至180 µg/周。有研究回顾性分析了3例BRAF突变的ECD患者，证明PEG-IFNα对于心脏、腹膜后肾脏周围、骨骼和中枢神经系统受累有效，但对眼眶肿物效果不佳[23]。

2. RAF抑制剂：

（1）维莫非尼（Vemurafenib）：BRAF抑制剂可作为IFN-α治疗失败或不耐受的二线治疗，也可作为心脏、中枢神经系统受累ECD患者的一线治疗。2013年，Haroche等[24]的研究应用维莫非尼治疗3例携带BRAF^V600E^突变的多系统受累难治性ECD患者，其中2例合并LCH，首次证明维莫非尼治疗ECD有效。另一项研究纳入8例BRAF^V600E^突变阳性的中枢神经系统或心脏受累ECD患者，予维莫非尼480 mg每日2次治疗，中位随访10.5个月，所有患者的症状均得到改善，所有患者均出现2～3级皮肤不良反应[25]。2017年发表的LOVE研究纳入了54例ECD患者，其中50例患者接受了维莫非尼治疗，75％的患者在停用维莫非尼后复发，中位复发时间为6个月，恢复治疗后所有患者的病情均有所改善，侧面证明维莫非尼可能需要长期维持治疗，但剂量不详[26]。另一项VE-BASKET Ⅱ期临床试验纳入了22例BRAF^V600E^突变的ECD患者，初始接受维莫非尼960 mg每日2次治疗，大部分患者因治疗过程中出现不良反应（关节痛、斑丘疹、疲劳、脱发、QT间期延长、皮肤乳头状瘤和角化过度）减量至480 mg每日2次，但减量后仍能维持疗效，1例患者因继发皮肤鳞状细胞癌停药。治疗总体缓解率为54.5％，完全缓解率为4.5％，FDG-PET/CT评估总体有效率达100％，随访2年无进展生存率为83％，总生存率为95％[27]。2017年11月6日美国食品和药品管理局（FDA）批准维莫非尼用于治疗BRAF^V600E^突变阳性的成人ECD患者。2020年ECD国际指南推荐维莫非尼作为心脏、中枢神经系统受累或伴终末器官功能障碍的BRAF^V600E^突变阳性ECD患者的一线治疗。2021年我中心回顾性分析了接受维莫非尼治疗的12例ECD患者，2例完全缓解，10例部分缓解，2年总生存率和无进展生存率分别为88.89％和66.67％[28]。

（2）其他RAF抑制剂：2013年首次报道达拉非尼（Dabrafenib）用于治疗BRAF^V600E^突变阳性的ECD患者，初始剂量为150 mg每日2次，后因出现2级关节痛剂量减半[29]。有研究发现，达拉非尼也会引起乏力、皮肤过度角化等不良反应。还有案例报道，达拉非尼可诱发胰腺炎[30]。但达拉非尼并未获批治疗ECD。

在相似浓度下，康奈非尼（Encorafenib）抑制BRAF^V600E^活性的解离半衰期较维莫非尼或达拉非尼延长10倍以上，故康奈非尼能持续抑制靶点、减少正常组织中MAPK通路的反常激活，减少不良反应。2021年报道了1例携带BRAF^V600E^突变的ECD患者应用康奈非尼100 mg/d治疗有显著反应，一个月后因不良反应（掌跖红细胞感觉异常综合征、肌痛、脱发）剂量减半[31]。

2017年还有个案报道索拉非尼（Sorafenib）治疗携带ARAF^S214A^突变的眼部受累ECD患者，10个月后患者双眼视力改善，视网膜炎症减轻[32]。

3. MEK抑制剂：MEK抑制剂也被作为IFN-α治疗失败或不耐受后的二线治疗。

考比替尼（Cobimetinib）是一种MEK1/MEK2抑制剂，已获批治疗ECD。2018年有研究报道了3例BRAF野生型ECD患者应用考比替尼40 mg/d治疗有效[33]。2019年Diamond等[34]研究了12例应用考比替尼治疗的ECD患者，总有效率为89％，最常见的不良反应是恶心、痤疮样皮疹和横纹肌溶解综合征。还有个案报道显示，MAP2K1突变的ECD患者应用考比替尼治疗8个月后皮肤和垂体病变完全消失[35]。

曲美替尼（Trametinib）是另一种MEK抑制剂，应用剂量为2 mg/d。有研究报道1例BRAF^V600E^突变的ECD患者应用达拉非尼治疗后疾病进展，随后在新出现的病灶中发现KRAS突变、BRAF野生型，该患者应用曲美替尼治疗后疾病缓解。提示在初始治疗出现耐药后应再次对新发病灶进行突变检测，同时应用BRAF/MEK抑制剂可能是未来治疗的方向[36]。另一项研究报道，达拉非尼联合曲美替尼治疗中枢神经系统受累的ECD，患者临床症状明显改善[37]。

4. mTOR抑制剂：mTOR抑制剂不推荐作为ECD的一线治疗，但可作为难治复发ECD患者的挽救性治疗选择。一项前瞻性Ⅱ期研究纳入了10例应用西罗莫司（Sirolimus）联合泼尼松治疗的ECD患者，80％的患者至少有一个疾病部位客观缓解，主要在心血管（75％）、腹膜后（62.5％）受累的患者中观察到治疗反应[38]。另一项研究纳入了20例ECD患者，其中15例患者应用西罗莫司2 mg/d，另5例患者应用依维莫司（Everolimus）0.75 mg每日两次，65％的患者有治疗反应，15％的患者病情控制稳定，仅1例患者因不良反应停止治疗[39]。

5. 抗细胞因子治疗：阿那白滞素（Anakinra）是一种重组白细胞介素-1（IL-1）受体拮抗剂，靶向作用于IL-1β。2010年有研究在2例ECD患者中应用阿那白滞素100 mg/d治疗有效，除注射部位局部反应外未出现其他不良反应[40]。2016年有研究报道，小剂量维莫非尼联合阿那白滞素治疗ECD患者取得显著效果，随访13个月病情稳定[41]。一项研究纳入了63例ECD患者，8例应用阿那白滞素治疗，总体有效率为50％，38％的患者出现血小板减少、肝酶升高等不良反应[42]。还有研究报道，阿那白滞素治疗心脏受累的ECD取得良好疗效。

英夫利昔单抗（Infliximab）是一种抗TNF-α人鼠嵌合型单克隆抗体，2012年首次报道其对2例ECD患者治疗有效。2014年有研究报道，应用英夫利昔单抗5 mg/kg每6周1次联合甲氨蝶呤20 mg每周1次治疗ECD患者，6个疗程后取得良好疗效[43]。2017年一项研究纳入了63例ECD患者，5例患者应用英夫利昔单抗治疗无效，2例出现输液反应、肝酶异常[42]。有研究报道，英夫利昔单抗对2例心脏受累的ECD患者治疗有效，可能是由于TNF-α与ECD患者的内皮屏障破坏有关。

托珠单抗（Tocilizumab）是一种靶向IL-6受体的拮抗剂，一项研究中3例ECD患者接受托珠单抗治疗，3年总有效率为67％[44]。还有文献报道，多发血栓栓塞、多器官受累的ECD患者应用托珠单抗治疗疗效显著。

6. 其他靶向治疗：有研究报道2例ECD患者应用酪氨酸激酶抑制剂伊马替尼治疗后1例出现治疗反应。另一项研究对6例多系统受累、常规治疗无效的ECD患者应用伊马替尼治疗，中位剂量350（100～800）mg/d，中位随访12.5个月后2例患者病情稳定，4例患者进展，未发现重大不良反应[45]。

2022年一项研究报道显示，1例CSF1R体细胞突变、神经系统受累的ECD患者应用CSF1R抑制剂培西达替尼（Pexidartinib）治疗有效[19]。

7. 糖皮质激素、化疗：2015年一项研究回顾性分析了448例ECD患者，其中超过72％的患者接受过糖皮质激素治疗，多例患者应用过化疗药物和免疫抑制剂，包括环磷酰胺（12％）、长春新碱（12％）、甲氨蝶呤（6％）、克拉屈滨（5％）、硫唑嘌呤（4％）、依托泊苷（3％）和环孢素A（2％）。随着靶向治疗的发展，造血干细胞移植已几乎被放弃。糖皮质激素对于轻症ECD患者控制症状、缓解水肿和疼痛有效，对中枢神经系统受累的重症患者治疗效果不佳。

传统化疗药物中，甲氨蝶呤单药应用或与泼尼松或其他生物制剂联合使用，仅对13例ECD患者中的3例有效。对于中枢神经系统受累患者，可采用中剂量阿糖胞苷治疗，不良反应包括血细胞重度减少、感染、恶心、呕吐等。本中心在2018年报道了一例BRAF野生型ECD患者在IFN-α治疗期间中枢神经系统复发，应用中剂量阿糖胞苷挽救性治疗获得显著疗效[46]。本中心2020年报道，2例中枢神经系统受累的ECD患者应用阿糖胞苷一线治疗获得良好的疗效[47]。克拉屈滨也被证实对治疗ECD有效。2017年一项研究纳入了21例接受克拉屈滨治疗的ECD患者，总体临床有效率为52％，总体放射反应率为54％，其中3例（18％）疾病稳定，5例（30％）疾病进展，治疗相关不良反应包括2例感染和2例血细胞减少[48]。

8. 手术及局部治疗：一项纳入448例ECD患者的回顾性研究发现，13％的患者接受过局部放疗，6％的患者接受过脑或骨骼的手术[49]。对缩窄性心包炎、中枢神经系统受累的ECD患者进行手术治疗可缓解症状。对于眼内受累的ECD患者可进行玻璃体内注射抗血管内皮生长因子或光动力治疗。

三、总结

ECD的突变谱系中，BRAF^V600E^突变占50％以上，此外还有MAP2K1、其他BRAF、ARAF、NRAS、KRAS、PIK3CA等突变，涉及MAPK通路和PI3K-mTOR通路，证明ECD是一种克隆性肿瘤性疾病。ECD预后较差，目前暂无治愈ECD的已知方案，ECD的一线治疗药物是IFN-α，维莫非尼、曲美替尼等针对BRAF或MEK的靶向治疗被证明有良好疗效，少见基因突变的发现为寻找新型靶向治疗提供了依据。
